# Efficiency of vanillin in impeding metabolic adaptability and virulence of *Candida albicans *by inhibiting glyoxylate cycle, morphogenesis, and biofilm formation 

**DOI:** 10.18502/cmm.6.1.2501

**Published:** 2020

**Authors:** Saibabu Venkata, Fatima Zeeshan, Ahmad Kamal, Ahmad Khan Luqman, Hameed Saif

**Affiliations:** 1 Amity Institute of Biotechnology, Amity University Haryana, Gurugram, India; 2 Department of Biosciences, Jamia Millia Islamia, New Delhi, India; 3 Department of Pharmaceutical Chemistry, Jamia Hamdard, New Delhi, India

**Keywords:** Biofilm, Caenorhabditis elegans, Candida, Glyoxylate cycle, Morphogenesis, Vanillin

## Abstract

**Background and Purpose:**

*Candida albicans* is the fourth most common cause of nosocomial fungal infections across the world. The current drug regimens are suffering from such drawbacks as drug resistance, toxicity, and costliness; accordingly, they highlight the need for the discovery of novel drug agents. The metabolic adaptability under low-carbon conditions and expression of functional virulence traits mark the success of pathogens to cause infection. The metabolic pathways, such as glyoxylate cycle (GC), enable *C. albicans *to survive under glucose-deficient conditions prevalent in the hostile niche. Therefore, the key enzymes, namely isocitrate lyase (ICL) and malate synthase (MLS), represent attractive agents against *C. albicans*. Similarly, virulence traits, such as morphogenesis and biofilm formation, are the crucial determinants of *C. albicans* pathogenicity. Regarding this, the present study was conducted to uncover the role of vanillin (Van), a natural food flavoring agent, in inhibiting GC, yeast-to-hyphal transition, and biofilm formation in human fungal pathogen *C. albicans*.

**Materials and Methods:**

For the determination of hypersensitivity under low-glucose conditions, phenotypic susceptibility assay was utilized. In addition, enzyme activities were estimated based on crude extracts while in-silico binding was confirmed by molecular docking. The assessment of morphogenesis was accomplished using hyphal-inducing media, and biofilm formation was estimated using calcofluor staining, MTT assay, and biomass measurement. Additionally, the in vivo efficacy of Van was demonstrated using *Caenorhabditis elegans* nematode model.

**Results:**

Based on the results, Van was found to be a potent GC inhibitor that phenocopied *ICL1* deletion mutant and displayed hypersensitivity under low-carbon conditions. Accordingly, Van facilitated the inhibition of ICL and MLS activities in vitro. Molecular docking analyses revealed the in-silico binding affinity of Van with *Icl*1p and *Mls*1p. Those analyses were also confirmative of the binding of Van to the active sites of both proteins with better binding energy in comparison to their known inhibitors. Furthermore, Van led to the attenuation of such virulence traits as morphogenesis, biofilm formation, and cell adherence. Finally, the antifungal efficacy of Van was demonstrated by the enhanced survival of *C. elegans* with *Candida* infection. The results also confirmed negligible hemolytic activity on erythrocytes.

**Conclusion:**

As the findings of the present study indicated, Van is a persuasive natural compound that warrants further attention to exploit its anticandidal potential.

## Introduction

*Candida albicans* is among the most common fungal microflora that resides in the mucocutaneous cavities of the skin, vagina, and intestine of humans as commensal organisms. However, they can turn pathogenic after the alteration of the immune system [1]. With a substantial rise in the number of immunocompromised patients, the potential risk implicated in the occurrence of fungal diseases has considerably been alarming [2]. Widespread use of the existing limited antifungals, as well as the impeding progress in the development of new antifungal drugs, has led to a rise in multidrug resistance [2].

Phytochemicals, in particular, those having proven beneficial properties and negligible toxicity, have become an immense source of interest to be exploited for their antifungal potential [3]. Vanillin (Van) or 4-hydroxy-3-methoxybenzaldehyde is one such natural food flavoring agent which has been granted “generally regarded as safe” status with an acceptable daily intake of 10 mg/kg as agreed between Food and Agriculture Organization/World Health Organization and European Union [4]. However, the antifungal potential of Van has been never elucidated.

Fitness attributes like metabolic flexibility and virulence traits (e.g., morphogenesis and biofilm formation) are the crucial determinants of the pathogenicity of *C. albicans *[5, 6]*. *Glyoxylate cycle (GC) is a widely studied metabolic pathway in many organisms, including *C. albicans*, *Aspergillus fumigatus*, *Mycobacterium tuberculosis,* and *Burkholderia *species [7-9]. Glyoxylate cycle acts as a significant metabolic bypass for tricarboxylic acid cycle to consume simple carbon (C_2_) compounds when glucose-deficient conditions are prevailing. This cycle, therefore, permits the utilization of C_2_ compounds and prevents the loss of two carbons by bypassing the steps of CO_2_ generation in tricarboxylic acid cycle, thereby facilitating the anaplerotic maintenance of intermediates. The key enzymes of GC, namely isocitrate lyase (ICL) and malate synthase (MLS), represent an attractive antifungal target since GC is absent in humans. Furthermore, *C. albicans*, lacking either *Icl*1 or *Mls*1, are less virulent in the mouse models of systemic candidiasis [9, 10]. 

Similarly, the expression of functional virulence traits, such as morphogenesis and biofilm formation, is also known to be crucial for pathogenicity [6]. *Candida albicans* exists in either rapidly dividing yeast or filamentous invasive hyphal form, which is the characteristic of filamentous fungi. Likewise, *Candida* biofilm structure is particularly difficult to eradicate since biofilm is much more resistant to antifungal agents than planktonic cells, and hyphal form is necessary to form a biofilm. With this background in mind, the aim of the present study was to evaluate the effect of Van on the metabolic adaptability, morphogenesis, and biofilm formation of *C. albicans*.

## Materials and Methods

**Growth conditions**

*Candida albicans* strains used in the study are mentioned in Table S1. The strains were cultured in YPD broth (Himedia, India) with the composition of yeast extract 1%, peptone 2%, and dextrose 2%. For agar plates 2%, agar was added to the media. The cells were freshly revived on YPD broth and then transferred to the agar plate. For GC analysis, the cells were grown in yeast nitrogen base (YNB; Himedia, India) with 0.67% YNB and 2% agar (for solid plates) supplemented with different carbon sources, including 2% glucose (CDH, India), 2% citrate (CDH, India), 2% acetate (CDH, India), 5% ethanol (CDH, India), and 2% glycerol (CDH, India). To check the persistence of *Candida *cells in *C. elegans*, the cells were grown on brain heart infusion (BHI) media (Himedia, India) and then were fed to *C. elegans*.

***Spot assay***

To check the phenotype susceptibility under glucose-deprived conditions, spot assay was performed as described previously in the absence (control) and presence of Van (Sigma Chemical Co., USA) [11]. Briefly, 5 μl of five-fold serially diluted yeast cultures (OD_600_ 0.1) was spotted onto YPD plates. The growth difference was measured after 48 h at 30°C.

***Isocitrate lyase and malate synthase enzyme assay***

The GC enzyme measurement was accomplished using our previously reported methods [11]. DL-Isocitric acid (HiMedia, India) and acetyl CoA (Sigma Chemical Co., USA) were used as the substrates for the ICL and MLS enzyme activities, respectively. In addition, glyoxylate-phenylhydrazone (HiMedia, India) and 5-thio-2-nitrobenzoic acid (HiMedia, India) formation was spectrophotometrically assessed at 324 and 412 nm for the ICL and MLS enzymes, respectively. 

**Docking studies**

AutoDock 4.2 package was used for the docking of Van with *Icl*1p and *Mls*1p [11- 14]. The interaction of *Icl*1p and *Mls*1p with Van was analyzed using the Lamarckian genetic algorithm. The binding energy was calculated using van der Waals, electrostatic interactions, and hydrogen bonding and then compared with their known counterparts, namely 3-nitro propionate and bromopyruvate, respectively. I-TASSER server was utilized to generate a 3D model of query sequence of *Icl*1p (PDB: 1DQU) and *Mls*1p (PDB: 3CUZ) by multiple threading alignments and iterative structural assembly simulation, respectively. Finally, the docked complexes of *Icl*1p and *Mls*1p were further optimized, validated, and explored using the “Protein-Ligand Interactions” modules of the Discover Studio (version 4.0).

***Morphogenesis***

Morphogenesis of *C. albicans* was carried out on hyphal induction media as described previously [15]. Briefly, the culture was grown overnight at 30^o^C in YPD broth. Subsequently, the revived cells were harvested by centrifugation at 5,000 rpm for 3 min, washed twice, and incubated at 37^o^C for 6 h with phosphate-buffered saline (PBS) to induce starvation. The cells were transferred to the indicated media for hyphal growth with or without Van. Hyphae were observed under a microscope after the incubation.

**Adherence to epithelial cells**

Cell adherence assay was performed as described previously [15]. Briefly, equal volumes of buccal epithelial cells (BECs) and yeast cells were mixed with Van and incubated for 2 h at 37^o^C and a pH of 7.0. After incubation, the cells were washed and added with 1 µL trypan blue (Invitrogen, USA) solution (0.4 %) and then examined under a light microscope.

**Biofilm formation**

*Candida *biofilms were checked on the polystyrene surface of 96-well plates as previously described [15]. In brief, 200 μl of the prepared cell suspensions of 1×10^5^ cells/ml was added to the selected wells and incubated at 37°C for 1 h. After the removal of non-adherent cells by PBS washing, a fresh YPD medium containing Van was added to the adherent cells, and the plates were incubated at 37°C for 24 h.

The effect of Van on the pre-formed biofilms was estimated using the MTT (HiMedia, India) reduction assay, as previously described [15]. For cell adherence assay, the primarily treated and non-treated cells were grown till OD_600_ 1.0. After washing, the adhered cells were directly quantified through the MTT assay. For biofilm biomass measurement, pre-weighed sterile silicone squares (1.5×1.5 cm) were incubated in the presence or absence of Van at 37°C for 60 h at 75 rpm agitation. Furthermore, in order to measure dry mass, the squares were washed in PBS and then dried and weighed. The total biomass of each biofilm was calculated by subtracting the weight of the pre-weighed silicon square.

**Candida infection in Caenorhabditis elegans model**

*Caenorhabditis elegans-C. albicans* assay was performed according to the previously reported method [11]. To this end, 60 nematodes from BHI plates were transferred on *C. albicans* lawns for 2 h. Subsequently, the nematodes were washed off from the plates with a screen medium (30 % BHI broth in M9 buffer). The suspension pre-infected with* C. elegans* (20 µL) was added to the wells of enzyme-linked immunosorbent assay plates. Subsequently, 80 µL of screen medium containing Van (125 µg/mL) was dispensed into the indicated well. The nematode survival was checked every day for 4 days. For toxicity assays, non-infected worms were pipetted into the single wells of 96-well plates containing M9 buffer; then, Van was added. In the next stage, the plates were incubated at 25°C for 4 days. The results are expressed as the percentage of alive or dead worms after incubation.

**Hemolytic activity assay**

The hemolytic activity of Van was evaluated by measuring the absorbance at 540 nm to determine the release of hemoglobin from a 4% suspension of erythrocytes [15]. The percentage of hemolysis was calculated as follows: 

Hemolysis (%)=[(A_sample_-A_blank_)/(A_Triton_-A_blank_)]×100 

**Statistical analysis**

All experiments were performed in triplicates (n=3). The results were reported as mean±standard deviation and analyzed using Student’s t-test. A p-value less than 0.05 was considered statistically significant.

***Ethical considerations***

All the experiments involving human specimens (i.e., oral epithelial cells and blood) were performed in accordance with the relevant guidelines and regulations. The samples were taken only after ensuring patients' awareness and obtaining written informed consent. The study was approved by the Research Ethics Committee of the Amity University, Haryana, India (reference no. AUH/EC/RP/2016/05).

## Results

**Phenocopying of Icl1 mutant and inhibition of i** **socitrate lyase and malate synthase** **enzyme activities by vanillin**

**Figure 1.**
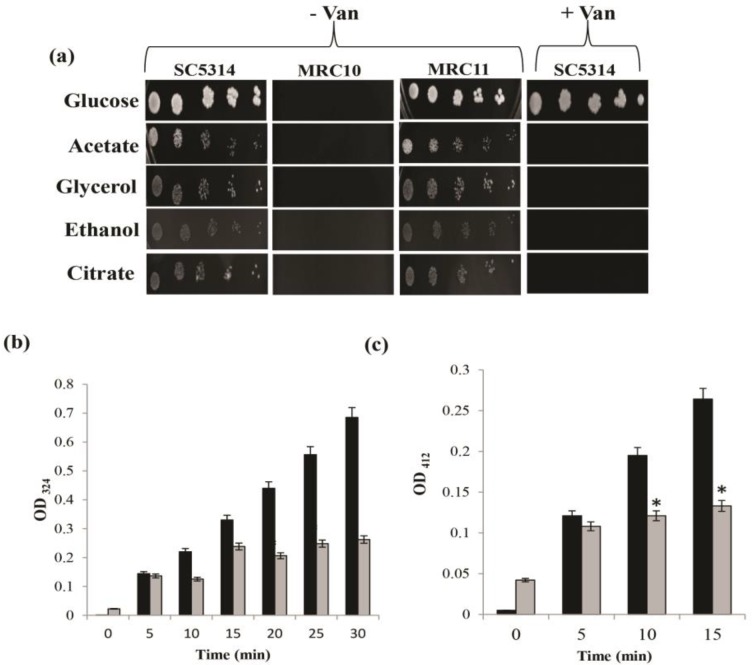
Effect of vanillin (Van) on glyoxylate cycle; (a) phenotypic susceptibility of Van to alternate carbon sources (*Candida albicans* strain SC5314 [wild-type], *Mrc*10 [Δicl1[, and *Mrc*11 [Δicl1+ICL1] were cultured on YNB agar plates containing the indicated carbon sources [2% glucose, 2% glycerol, 2% ethanol, 2% sodium citrate, and 2% sodium acetate] with or without Van for 2 days at 30°C), (b) *Icl*1p enzyme activity assay in the presence of Van (mean enzyme activity expressed as absorbance at 324 nm +SD is depicted on Y-axis), and (c) *Mls*1p enzyme activity in the presence of Van (mean enzyme activity expressed as absorbance at 412 nm +SD is depicted on Y-axis. Each data point represents the mean of three experiments. Black and grey bars respectively represent control and Van for the entire panels.)

Spot assay on different low-carbon sources, such as glycerol, ethanol, citrate, and acetate, revealed that *Icl*1 mutant (Δicl1) was hypersensitive to all the tested low-carbon conditions. In addition, the growth was similar to wild type in the revertant of *Icl*1 strain (Δicl1+ICL1; Figure 1a). The addition of Van-phenocopied Δ*Icl*1 resulted in similar hypersensitivity (Figure 1a). The results of in vitro enzyme assays indicated the role of Van in the inhibition of the activities of both *Icl*1p (Figure 1b) and *Mls*1p by more than 50% (Figure 1c). 

***Docking studies regarding the inhibitory effect of vanillin on*** ***isocitrate lyase and malate synthase*** ***enzymes***

**Figure 2:**
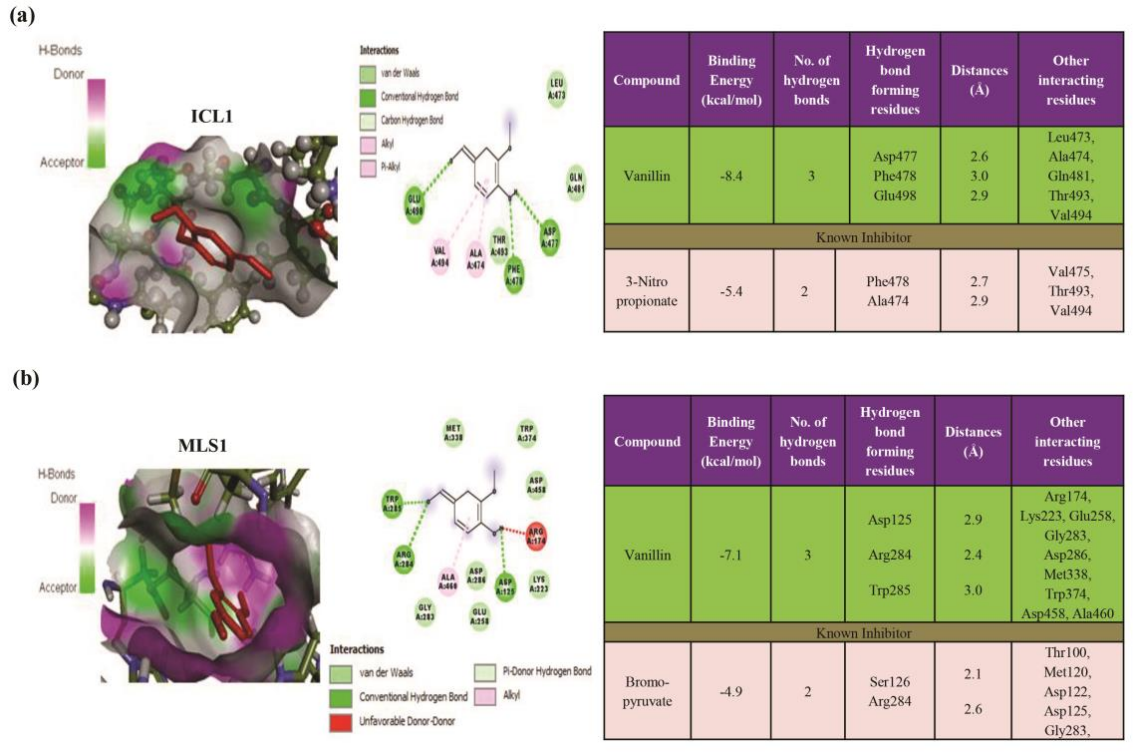
Molecular docking of vanillin (Van) with *Icl*1p and *Mls*1p; (a) surface view of *Icl*1p protein with Van (left panel) (the 2D schematic diagram shows the interactions of compound Van with the *Icl*1 [middle panel], as well as the binding energy and specific interaction of *Icl1*p with Van [right panel]), (b) surface view of *Mls*1p protein with Van (left panel) (2D schematic diagram shows the interactions of Van with *Mls*1p [middle panel], as well as the binding energy and specific interaction of *Mls*1p with Van [right panel]. Residues involved in hydrogen bonding, Van der Waals, carbon-hydrogen, and Pi-alkyl interactions are represented in different colors as indicated in the inset.)

AutoDock (version 4.2) was used to determine the orientation of inhibitors bound in the active sites of *Icl*1p and *Mls*1p. The results revealed that Van bound more efficiently into the active sites of ICL and MLS with a minimum binding energy (∆G) of -8.4 kcal/mol as compared to 3-nitropropionate (∆G) as a known inhibitor (-5.4 kcal/mol; Figure 2a). The conformation with the binding energy value for each molecule was chosen for further analysis. The results of these studies are displayed for *Icl*1p and *Mls*1p in figures 2a and 2b, respectively. The binding modes of *Icl*1p and *Mls*1p inhibitors were investigated using the Discover Studio (version 4.0). 

The Van forms a complex with *Icl*1p by offering three H-bonds among *Asp*477, *Phe*478, and *Glu*498 at 2.6, 3.0, and 2.9 Å, respectively. Similarly, Van was observed to bind to the active site of *Mls*1p with a minimum binding energy (∆G) of -7.1 kcal/mol as compared to bromopyruvate, as a known inhibitor, showing a minimum binding energy (∆G) of -4.9 kcal/mol (Figure 2b). The Van offered three hydrogen bonds among *Asp*125 (2.9 Å), *Arg*284 (2.4 Å), and *Trp*285 (3.0 Å) (Figure 2). In addition, the analysis of docked structures showed that *Icl*1p and *Mls*1p generated numerous Van der Waals, covalent, carbon hydrogen, Pi alkyl, and electrostatic interactions with Van. 

**Figure 3.**
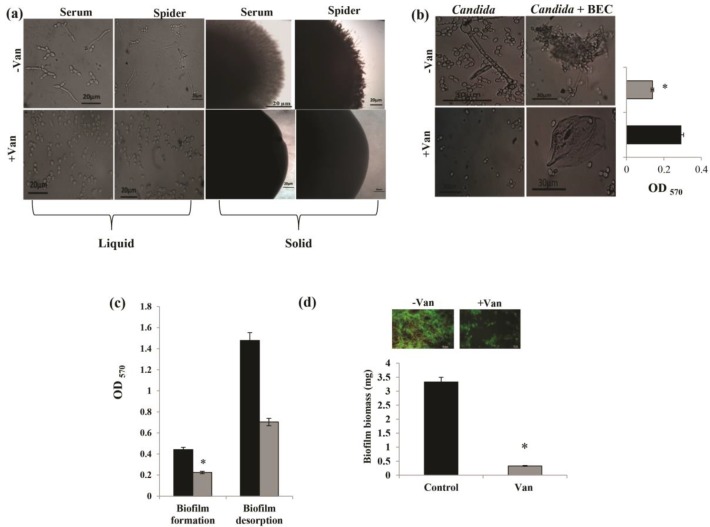
Effect of vanillin (Van) on virulence traits; (a) left panel showing hyphal morphogenesis in the liquid hyphal-inducing media (YPD with 10% serum and Spider media) in the absence (control) and presence of Van (Magnification 40X) and right panel presenting hyphal morphogenesis in the solid hyphal-inducing medium (10% serum and Spider agar medium) in the absence (control) and presence of Van (magnification 4X), (b) effect of Van on the adherence of *Candida* to epithelial cells (left panel depicts untreated cells forming pseudohyphae [left] and adhering to BECs [right], and the lower panel displays the treated cells existing only in yeast form [left] and not adhered to BECs [right]. The right panel depicts the bar graph of mean cell adherence to microtitre polystyrene surface which is quantified by MTT assay and depicted as the mean of OD_450_ nm ±SD of three independent sets of experiments illustrated on Y-axis. * depicts *P<0.05*), (c) effect of Van on the biofilm formation and biofilm desorption of *C. albicans* depicted as bar graph and quantified by using MTT assay (mean of OD_450_ nm ±SD of three independent sets of experiments are depicted on Y-axis, and * depicts *P<0.05*), (d) effect of Van on biofilm biomass formed on silicone sheets (mean of dry weight ±SD of three independent sets of experiments are depicted on Y-axis, and * depicts *P<0.05*. Inset depicts the fluorescence microscopic images of CFW-stained biofilms.)

**Inhibition of morphogenesis and biofilm formation in Candida albicans by vanillin**

Our results revealed that Van (62.5 µg/mL)-treated cells were unable to express filaments in both liquid and solid sera and Spider media contrary to the untreated cells under various hyphal-inducing conditions at 37^o^C (Figure 3a). An adherence assay was performed using BECs to clarify if inhibited yeast-to-hyphal transition could affect the adherence of *Candida* to the epithelial cells. The *C. albicans* treated with Van (62.5 µg/mL) presented a normal morphology with few or no adherence to BECs. On the contrary, in the controls, *C. albicans* presented as pseudohyphae adhering to BECs with only a few cells found free in the culture medium (Figure 3b). 

Further quantification by MTT assay showed that the adherence of *Candida* cells to polystyrene surface was reduced by 52% (Figure 3b). In addition, the estimation of biofilm formation by MTT assay confirmed the reduction of biofilm formation by 49% in the presence of Van (Figure 3c). In addition, a 52% mature biofilm eradication was observed in the presence of Van (Figure 3c). The decreased biofilm biomass confirmed the inhibitory effect of Van against biofilm formation (Figure 3d).

**Inhibition of persistence of Candida elegans-infected with Candida albican species by vanillin**

**Figure 4:**
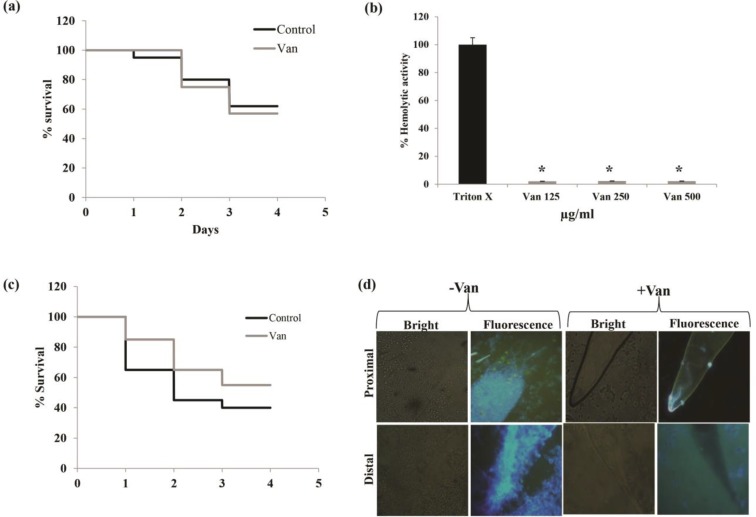
Effect of Van (vanillin) on *Candida elegans* survival; (a) Kaplan-Meier curve showing the survival percentage of *C. elegans* in the presence of Van (the toxicity of Van was studied on nematodes by determining survival rates for 4 days), (b) hemolytic activity of Van depicted as percentage on Y-axis in comparison to that of the positive control Triton X 100, (c) Kaplan-Meier curve showing the survival percentage of *C. albicans*-infected *C. elegans* in the presence of Van, (d) intestinal persistence of *C. albicans* (CFW-stained *C. albicans* were visualized in the proximal and distal intestinal regions of the *C. elegans* treated with Van. Black and grey bars respectively represent control and Van for entire panels.)

The toxicity of Van was examined by treating worms for 4 days in the absence of *Candida *infection. The results suggested that Van had no toxic effects on nematode (Figure 4a). Additionally, the hemolytic activity of Van was tested against erythrocytes. The results revealed less than 10% hemolysis even at higher concentrations of Van in comparison to that observed for Triton X (Figure 4b). It was also observed that Van protected *C. elegans* against *C. albicans* infection and enhanced its survival (Figure 4c). This was also reflected from the persistence assay performed by calcoflour white (CFW) staining where *C. albicans *cells were clearly visible in the proximal and distal intestinal regions of the untreated* C. elegans, *which were absent or negligible in Van-treated worms (Figure 4d).

## Discussion

With the aim of uncovering the unconventional medications that possess antifungal properties, the present study was conducted to explore the antifungal potential of Van, a naturally occurring food flavoring agent, against *C. albicans*. The GC is one of the key cycles, which enables *C. albicans* to survive in a wide variety of glucose-depleted niches to establish candidiasis. Recently, the GC cycle was found to be essential for the virulence of several other pathogens, and it has been explored as a potential drug target since GC is absent in humans [16]. 

Several observations, such as phenocopying of *Icl*1 mutant by Van treatment under glucose-limiting conditions (Figure 1a) and reduced activities of *Icl*1p (Figure 1b) and *Mls*1p (Figure 1c) enzymes, project Van as a potential GC inhibitor and deserve attention. Moreover, these results were consistent with those of the docking studies, revealing the binding of Van to the active sites of these enzymes (Figure 2). Our findings highlighted that Van yielded a preeminent dock score for ICL and MLS enzymes. Protein-ligand interactions accentuated that the van der Waals, covalent, carbon-hydrogen, Pi alkyl, and electrostatic interactions made a ruling contribution at the active site. 

Molecular docking operation distinguishes the foremost docking binding energy value against these receptor molecules. Deliberated binding from molecular docking yielded the minimum energy values of -8.4 and -7.1 kcal/mol for Van with proteins *Icl*1p and *Mls*1p, respectively, compared to their known inhibitors. Nevertheless, this study introduced a novel class of multitarget Van compound. Based on the bioinformatics approach, it was established that Van showed excellent binding energy for ICL and MLS, and it may be considered a good inhibitor of GC enzymes.

This study also involved the assessment of the effect of Van on morphogenesis which is a key virulence attribute of *C. albicans*. Two different hyphal-inducing media were used in the study, and it was observed that Van was able to inhibit filamentation in both serum and Spider media (Figure 3a). Adherence of *C. albicans* to the mucosal epithelial cells is considered as the initial step in the mucocutaneous candidiasis. Our results revealed the reduced adherence of *Candida* cells to BECs (Figure 3b). Conceptualization of *Candida *host cell interaction will have important implications for figuring out the therapeutic strategies of candidiasis. 

Biofilm formation is another significant virulence trait, apart from the morphogenetic switching. Moreover, functional hypha is a prerequisite for biofilm formation and known to mediate the dissemination of *C. albicans* to the host tissues by invasion. Based on the evidence, the virulence of *C. albicans *is reduced in hypha-deficient mutants. This issue emphasizes the importance of hypha formation in *C. albicans *infection [17]. The formation of *C. albicans* biofilms could enhance the resistance of this pathogen to most of the commonly used antifungal agents. 

The aforementioned facts prompted us to closely examine the effect of Van on the biofilm formation of *C. albicans.* Our results were indicative of the significant effect of Van against *C. albicans* biofilms not only at the formation step but also at the mature stage, leading to biofilm desorption (Figure 3c). Inhibited biofilm formation was also substantiated by reduced biofilm biomass in the presence of Van (Figure 3d). These observations suggested Van as a potent inhibitor of *C. albicans* virulence traits.

Lastly, an in-vivo study was conducted on *C. elegans, *a widely used nematode, to validate the effectiveness of Van in *Candida* infection. *Candida elegans* has been successfully used as a candidiasis infection model for the identification of new antifungal compounds [18, 19]. Nematodes consume fungal pathogens and establish an infection within the worm gut that can be identified for yeast accumulation inside *C. elegans.* Regarding this, in the current study, *C. elegans* was employed to substantiate the antifungal effect of Van. It was confirmed that in the presence of Van, the survival rate of *C. elegans* was markedly increased by preventing the growth of *C. albicans* (Figure 4c). 

After staining the *C. albicans *cells with CFW, which specifically stains chitin present only in the yeast cell wall, they were clearly visible in the proximal and distal intestinal regions of the untreated* C. elegans. *However, they were absent or negligible in the Van-treated worms (Figure 4d). Similar results were reported in earlier studies where nematodes were treated with essential oil α-longipinene [20]. This implies that these new antifungal agents are effective in treating worms infected with *Candida*. 

## Conclusion

In conclusion, given the ability of Van to target GC and block significant virulence traits as demonstrated in the present study, immediate attention is warranted for further studies to enable Van to be used as an effective medicine for the treatment of *Candida* infections.

## References

[1] Pfaller MA, Diekema DJ (2007). Epidemiology of invasive candidiasis: a persistent public health problem. Clin Microbiol Rev.

[2] Prasad R, Kapoor K (2005). Multidrug resistance in yeast Candida. Int Rev Cytol..

[3] Saibabu V, Fatima Z, Khan LA, Hameed S (2015). Therapeutic potential of dietary phenolic acids. Adv Pharmacol Sci..

[4] World Health Organization (2004). Summary of evaluations performed by the joint FAO/WHO expert committee on food additives.

[5] Fleck CB, Schöbel F, Brock M (2011). Nutrient acquisition by pathogenic fungi: nutrient availability, pathway regulation, and differences in substrate utilization. Int J Med Microbiol.

[6] Van Schaik EJ, Tom M, Woods DE (2009). Burkholderia pseudomallei isocitrate lyase is a persistence factor in pulmonary melioidosis: implications for the development of isocitrate lyase inhibitors as novel antimicrobials. Infect Immun.

[7] Ebel F, Schwienbacher M, Beyer J, Heesemann J, Brakhage AA, Brock M (2006). Analysis of the regulation, expression, and localisation of the isocitrate lyase from Aspergillus fumigatus, a potential target for antifungal drug development. Fungal Genet Biol.

[8] Krátký M, Vinšová J (2013). Advances in mycobacterial isocitrate lyase targeting and inhibitors. Curr Med Chem.

[9] Lorenz MC, Fink GR (2002). Life and death in a macrophage: role of the glyoxylate cycle in virulence. Eukaryotic Cell.

[10] Lorenz MC, Fink GR (2001). The glyoxylate cycle is required for fungal virulence. Nature.

[11] Ansari MA, Fatima Z, Ahmad K, Hameed S (2018). Monoterpenoid perillyl alcohol impairs metabolic flexibility of Candida albicans by inhibiting glyoxylate cycle. Biochem Biophys Res Commun.

[12] Webb W, Sali A (2017). Protein structure modeling with MODELLER. Methods Mol Biol..

[13] Wu S, Skolnick J, Zhang Y (2007). Ab initio modeling of small proteins by iterative TASSER simulations. BMC Biol..

[14] Ahmad K, Bhat AR, Athar F (2017). Pharmacokinetic evaluation of callistemon viminalis derived natural compounds as targeted inhibitors against δ-opioid receptor and farnesyl transferase. Lett Drug Design Discovery.

[15] Saibabu V, Fatima Z, Ahmad K, Khan LA, Hameed S (2019). Octyl gallate triggers dysfunctional mitochondria leading to ROS driven membrane damage and metabolic inflexibility along with attenuated virulence in Candida albicans. Med Mycol..

[16] Ebel F, Schwienbacher M, Beyer J, Heesemann J, Brakhage AA, Brock M (2006). Analysis of the regulation, expression, and localisation of the isocitrate lyase from Aspergillusfumigatus, a potential target for antifungal drug development. Fungal Genet Biol.

[17] Berman J, Sudbery PE (2002). Candida albicans: a molecular revolution built on lessons from budding yeast. Nat Rev Genet.

[18] Singulani JL, Scorzoni L, Gomes PC, Nazaré AC, Polaquini CR, Regasini LO, Fusco-Almeida AMMendes-Giannini MJS (2017). Activity of gallic acid and its ester derivatives in Caenorhabditis elegans and zebrafish (Danio rerio) models. Future Med Chem.

[19] Okoli I, Coleman JJ, Tampakakis E, An WF, Holson E, Wagner F (2009). Identification of antifungal compounds active against Candida albicans using an improved high-throughput Caenorhabditis elegans assay. PLoS One.

[20] Manoharan RK, Lee JH, Kim YG, Kim SI, Lee J (2017). Inhibitory effects of the essential oils alphalongipinene and linalool on biofilm formation and hyphal growth of Candida albicans. Biofouling.

